# Research participation after terrorism: an open cohort study of survivors and parents after the 2011 Utøya attack in Norway

**DOI:** 10.1186/s13104-016-1873-1

**Published:** 2016-02-01

**Authors:** Lise Eilin Stene, Grete Dyb

**Affiliations:** Norwegian Centre for Violence and Traumatic Stress Studies, NKVTS, Gullhaugveien 1-3, 0484 Oslo, Norway; Department of Social Pediatrics, Women and Children’s Division, Oslo University Hospital, P.b. 4956, Nydalen, 0424 Oslo, Norway; Institute of Clinical Medicine, Faculty of Medicine, University of Oslo, Oslo, Norway

**Keywords:** Selection bias, Disasters, Mass casualty incidents, Methods, Data collection, Research design

## Abstract

**Background:**

Reliable estimates of treatment needs after terrorism are essential to develop an effective public health response. More knowledge is required on research participation among survivors of terrorism to interpret the results properly and advance disaster research methodology. This article reports factors associated with participation in an open cohort study of survivors of the Utøya youth camp attack and their parents.

**Methods:**

Overall, 490 survivors were invited to two semi-structured interviews that were performed 4–5 and 14–15 months after the attack. The parents of 482 survivors aged 13–32 years were eligible for a complementary study. The study had an open cohort design in which all of the eligible survivors were invited to both waves. Pearson’s Chi squared tests (categorical variables) and independent t tests (continuous variables) were used to compare survivors by participation.

**Results:**

Altogether, 355 (72.4 %) survivors participated: 255 in both waves, 70 in wave 1 only, and 30 in wave 2 only. Compared with the two-wave participants, wave-1-only participants were more often non-Norwegian and reported higher exposure, whereas wave-2-only participants reported more posttraumatic stress, anxiety/depression, and somatic symptoms. In total, 331 (68.7 %) survivors had ≥1 participating parents, including 311 (64.5 %) with maternal and 243 (50.4 %) with paternal participation. Parental non-participation was associated with non-Norwegian origin, somatic symptoms and less social support. Additionally, paternal non-participation was associated with having divorced parents, and maternal non-participation was associated with higher age, not living with parents, posttraumatic stress and anxiety/depression symptoms.

**Conclusions:**

Survivors with initial non-participation had more symptoms than did the other participants. Thus, an open cohort design in post-terrorism studies might improve the participation among survivors with higher morbidity. Because the factors associated with maternal and paternal participation differed, it is important to consider potential disparities in the selection of mothers and fathers when interpreting parental data.

**Electronic supplementary material:**

The online version of this article (doi:10.1186/s13104-016-1873-1) contains supplementary material, which is available to authorized users.

## Background

A terrorist attack may cause serious mental and physical health problems, and reliable estimates of subsequent morbidity and treatment needs are essential to develop an effective public health response [1]. However, the unpredictable and chaotic circumstances of a terrorist attack make it difficult to conduct methodologically solid research. Previous post-disaster studies have often lacked a clear definition of the study population and not reported a participation rate [2, 3]. In addition, longitudinal studies have commonly failed to describe the characteristics of attrition [4]. Therefore, little is known regarding research participation among individuals who survive terrorism. Typically, research respondents have a higher socioeconomic status and better health than non-respondents do [5, 6]. Terror-related experiences may also influence the willingness to participate in research [4, 7]. On the one hand, the most severely affected survivors might be less likely to participate because of impaired function or fear of being reminded of the attack. On the other hand, such survivors might be more motivated to participate to increase our understanding of the adverse effects of terrorism. More knowledge is required regarding research participation after acts of terrorism to interpret the results properly and strengthen future study methods.

This article covers participation in two waves of an open cohort study of survivors of the Utøya attack and their parents. On July 22, 2011, a solitary perpetrator executed two terrorist attacks in Norway. After detonating a bomb in the Oslo Government Quarter, he committed a shooting massacre at the summer camp of the Norwegian Labour Party’s youth organisation on the Utøya islet. Overall, 564 persons were isolated on the islet during a 1.5-hour-long shooting; 69 were killed, and many were injured or risked drowning trying to escape by swimming. The shooting is considered a severe trauma because the victims were young, designated targets, and many were killed or injured or lost close ones [8, 9].

The aim of this study was to gain insight into research participation among survivors of terrorism and thereby advance disaster research methodology. Our specific objectives were to (a) identify the factors associated with participation in an open cohort study on survivors of the Utøya attack and (b) assess the characteristics of the survivors based on parental participation in a complementary study on the survivors’ parents.

## Methods

Overall, 495 survivors who had been on the Utøya islet during the shooting were identified from police records. The survivors resided in rural and urban municipalities across all Norwegian counties. The recruitment consisted of three stages: (1) a postal invitation, (2) a telephone call, and (3) an interview with those who received the call and agreed to participate. Postal study invitations were sent to 490 survivors. In the invitation letter, the survivors received information about the aims of the study, the study procedures, and an overview of the content and duration of the interview. Furthermore, they were informed that they could withdraw from the study at any time, and the letter provided contact information in case they had questions regarding the study or did not want to be contacted by phone. Four survivors aged <13 years and one survivor who lived abroad were excluded. The four survivors under 13 years of age were excluded because of their young age and the fact that they were mainly children accompanying leaders/guards. Moreover, the interview questionnaires were designed for adolescents and young adults. The study had an open cohort design in which all of the eligible survivors were invited to both waves. Semi-structured face-to-face interviews were performed at 4–5 months (wave 1) and 14–15 months (wave 2) after the shooting. The interviews included open-ended questions on several themes; such as their experiences during the shooting, the police interrogation, the trial, the return to school, and their use of social media, in addition to a wide range of topics assessed with closed-ended questions [10]. The respondents could choose between being interviewed at home or being interviewed at a location arranged by the interviewer. When the interview was completed, the respondents completed a questionnaire. If a respondent was unable to respond to the interview in Norwegian or English, it was proposed to perform the interview using an interpreter. The parents of 482 survivors aged 13–32 years were eligible for participation in a parallel study focusing on the reactions and experiences of the survivors’ parents. The current study assessed survivor reports only. A separate postal invitation addressed to parents/guardians of the survivor was sent to the addresses of the survivors. Next, the survivors were asked for the contact information of their parents during a telephone call. Parents of survivors born 1992 or later were eligible to participate by interview. Due to limited resources, parents of survivors born before 1992 were invited to participate by postal questionnaire. Reminders were sent to parents who did not answer the questionnaire on the first request.

### Variables

Our sociodemographic data included age, gender, country of origin, and whether the survivors lived with their parents, had divorced parents, were financially disadvantaged or resided in a peripheral municipality. Age was measured at the time of the attack as a continuous variable in years with one decimal. Non-Norwegian origin was defined as having both parents born abroad. Survivors were asked how they perceived their parents’ (those who lived with parents) or their own (those who did not live with parents) financial well-being compared with that of others. There were five response alternatives, which were dichotomized into financially disadvantaged (i.e., much or somewhat poorer) or not (i.e., similar, somewhat better, and much better). Peripheral residence described the location of the survivor’s home municipality at wave 1 in relation to communities of a certain size, in accordance with Statistics Norway’s centrality classification [11]. Municipalities located more than 45 min travelling time from communities with at least 15,000 inhabitants were classified as peripheral. We obtained information for all survivors on age, gender and place of residence from police records and on admittance to somatic hospitals directly after the attack from hospital records. The terror exposure assessment was explicitly designed to cover 13 potentially traumatic events experienced during the attack and has been demonstrated to be independently associated with mental health problems [12]. In the analyses, the mean sum score was applied. Posttraumatic stress reactions in the past month were assessed using the University of California at Los Angeles Post-traumatic Stress Disorder (PTSD) Reaction Index (UCLA PTSD-RI) [13]. The total score includes 17 items that conform to the 17 DSM–IV symptoms of PTSD rated on a 5-point Likert scale that ranges from 0 (never) to 4 (most of the time) [14]. Three items have two alternative wordings that are valued by the item with the highest score. The mean scores of the 17 items were used in the analyses. Cronbach’s alpha was 0.89 (wave 1 and 2).

Symptoms of anxiety and depression were measured with the Hopkins Symptom Checklist-8 (SCL-8), which is a short version of the SCL-25 [15]. It measures symptoms of depression/anxiety that occurred during the preceding 2 weeks using eight items scored on a scale from 1 (not bothered) to 4 (very much bothered). The mean score was applied in the analyses, and Cronbach’s alphas were 0.86 (wave 1) and 0.90 (wave 2). The short versions of the Hopkins Symptom Checklist have displayed high psychometric qualities in population-based studies [16]. Somatic symptoms that occurred during the preceding 2 weeks were assessed using a short version of the Children’s Somatic Symptoms Inventory (CSSI-8) [17]. The eight items assessed pain in the stomach, head, lower back, and arms/legs; faintness/dizziness; rapid heartbeat; nausea/stomach problems; and weakness. Each item was scored on a scale from 1 (not bothered) to 4 (very much bothered). The mean score was used in the analyses. Cronbach’s alphas were 0.77 (wave 1) and 0.78 (wave 2). Self-perceived social support was appraised using seven items from the Duke University of North Carolina Functional Social Support Questionnaire (FSSQ-7) and scored on a scale from 1 (much less than I would like) to 5 (as much as I would like) [18]. Mental health service (MHS) utilization was measured with a question on contact with specialized mental health services (yes/no). Wave 1 covered MHS utilization since the attack until wave 1 (ca. 0–5 months post-disaster). Wave 2 covered utilization from January 1, 2012, until wave 2 (ca. 5–15 months post-disaster).

### Ethics

Participants aged 16 years or older provided written informed consent. Written parental consent was required before survivors younger than 16 years old could participate in the study, as stipulated by Norwegian law. The interviewers were health practitioners who had received training in conducting research interviews of traumatised persons at a one-day seminar. The interviewers were instructed to offer help with contacting suitable services if they identified unmet needs. They worked in teams of two, and after each survey wave, they were invited to a one-day meeting to share experiences. In addition, a telephone line was provided for the interviewers where they could discuss the challenges that they encountered during the interviews and receive support. Most interviews were conducted at the homes of the respondents. If not, all travel expenses were covered. The participants did not otherwise receive financial compensation for study participation. After each survey wave, a brief summary of the initial results was sent to the respondents before the findings were published or reported to the media. The study was commissioned by the Norwegian Directorate of Health, and performed by the Norwegian Centre for Violence and Traumatic Stress Studies (NKVTS). The study was approved by the Regional Committees for Medical and Health Research Ethics South East and North in Norway, and the Norwegian police granted permission to access a list of the survivors’ names.

### Statistics

Pearson’s Chi squared tests (categorical variables) and independent t tests (continuous variables) were used to compare survivors by participation as follows. The exact test was used if the expected count was less than five for categorical variables. Survivors who participated in ≥1 waves were compared with non-participants (Table 1). Survivors who participated in two waves were compared with participants in one wave only (Table 2). Survivors with parental participation were compared with survivors without parental participation (Table 3). The reported percentages were based on the total number of answers for each item. We applied a two-sided statistical significance level of 0.05. The analyses were conducted using IBM SPSS version 20.0.

**Table 1:** Characteristics of survivors according to participation in ≥1 study waves (n = 490)

Characteristics of survivors	Non-participants (n = 135)		Participants (n = 355)		p value
	n/mean	(%/sd)	n/mean	(%/sd)	
Mean age in years	19.0	(3.9)	19.3	(4.6)	0.473
Male gender	83	(61.5)	184	(51.8)	0.055
Residing in peripheral municipality	24	(17.8)	50	(14.3)	0.338
Hospitalized	8	(5.9)	28	(7.9)	0.457
Any parental participation	27	(20.0)	304	(85.6)	<0.001
Maternal participation	22	(16.3)	289	(81.4)	<0.001
Paternal participation	15	(11.1)	228	(64.2)	<0.001

**Table 2:** Characteristics of survivors according to participation in one or both survey waves

Characteristics	Wave 1					Wave 2				
	Participated in wave 1 and 2 (n = 255)		Participated in wave 1 only (n = 70)		p value	Participated in wave 1 and 2 (n = 255)		Participated in wave 2 only (n = 30)		p value
	n/mean	(%/sd)	n/mean	(%/sd)		n/mean	(%/sd)	n/mean	(%/sd)	
Mean age at attack (years)	19.4	(4.3)	19.3	(5.7)	0.887	19.4	(4.3)	18.8	(4.1)	0.444
Male gender	139	(54.5)	33	(47.1)	0.274	139	(54.5)	12	(40.0)	0.132
Non-norwegian origin	25	(9.9)	14	(20.3)	0.019	25	(9.9)	2	(7.4)	0.757
Financially disadvantaged	51	(20.5)	17	(25.4)	0.387	51	(20.5)	6	(21.4)	0.906
Living with parent(s)	154	(61.1)	48	(70.6)	0.151	118	(47.0)	13	(44.8)	0.823
Divorced parents	96	(38.9)	30	(44.8)	0.381	96	(38.7)	13	(44.8)	0.523
Youth Labour Party member	234	(94.4)	61	(87.1)	0.040	234	(94.4)	27	(96.4)	0.723
Sibling(s) in the study	27	(10.6)	5	(7.1)	0.391	27	(10.6)	2	(6.7)	0.561
Hospitalized	19	(7.5)	5	(7.1)	0.930	19	(7.5)	4	(13.3)	0.281
Terror exposure (mean 0–13)	8.28	(2.24)	9.32	(1.91)	<0.001	8.28	(2.24)	9.05	(2.00)	0.103
Posttraumatic stress (mean PTSD-RI)	1.53	(0.72)	1.67	(0.68)	0.164	1.21	(0.67)	1.60	(0.77)	0.003
Anxiety/depression symptoms (mean SCL-8)	2.05	(0.66)	2.16	(0.64)	0.204	1.77	(0.62)	2.11	(0.86)	0.043
Somatic symptoms (mean CSSI-8)	1.72	(0.54)	1.73	(0.53)	0.944	1.62	(0.48)	1.90	(0.67)	0.033
Social support (mean FSSQ-7)	4.56	(0.57)	4.56	(0.59)	0.951	4.56	(0.60)	4.53	(0.61)	0.753
Mental health service utilization	180	(72.0)	54	(77.1)	0.391	169	(67.3)	23	(79.3)	0.188

Wave 1 (n = 325) was performed 4–5 months after the attack; wave 2 (n = 285) was performed 14–15 months after the attack

**Table 3:** Survivor characteristics by maternal and paternal participation among survivors aged 13–32 years who participated in wave 1 or 2 (n = 348)

Survivor characteristics		Paternal participation					Maternal participation					Any parental participation				
		Yes (n = 228)		No (n = 120)		p value	Yes (n = 289)		No (n = 59)		p value	Yes (n = 304)		No (n = 44)		p value
		n/mean	(%/sd)	n/mean	(%/sd)		n/mean	(%/sd)	n/mean	(%/sd)		n/mean	(%/sd)	n/mean	(%/sd)	
Mean age in years		18.72	(2.74)	19.17	(3.85)	0.263	18.62	(2.84)	20.15	(4.24)	0.010	18.59	(2.82)	20.82	(4.54)	0.003
Male gender		118	(51.8)	63	(52.5)	0.895	148	(51.2)	33	(55.9)	0.508	154	(50.7)	27	(61.4)	0.184
Non-norwegian origin		14	(6.1)	25	(21.7)	<0.001	19	(6.6)	20	(37.0)	<0.001	23	(7.6)	16	(41.0)	<0.001
Financially disadvantaged		42	(19.2)	29	(24.6)	0.246	56	(20.0)	15	(26.3)	0.286	59	(20.1)	12	(27.9)	0.239
Divorced parents (wave 1)		72	(34.0)	53	(54.6)	0.001	112	(42.4)	13	(28.9)	0.087	116	(41.9)	9	(28.1)	0.133
Living with ≥ 1 parents (wave 1)		138	(65.7)	64	(61.5)	0.467	179	(67.3)	23	(47.9)	0.010	188	(67.4)	14	(40.0)	0.001
Sibling(s) in the study		29	(12.7)	5	(4.2)	0.011	31	(10.7)	3	(5.1)	0.183	34	(11.2)	0	(0.0)	0.025
Hospitalized		17	(7.5)	11	(9.2)	0.577	25	(8.7)	3	(5.1)	0.442	27	(8.9)	1	(2.3)	0.152
Terror exposure (mean 0–13)		8.53	(2.22)	8.62	(2.21)	0.724	8.52	(2.16)	8.74	(2.50)	0.510	8.53	(2.17)	8.74	(2.54)	0.580
Mental health service utilization	Wave 1	152	(71.7)	76	(74.5)	0.601	190	(71.7)	38	(77.6)	0.399	200	(71.9)	28	(77.8)	0.460
	Wave 2	119	(65.0)	67	(73.6)	0.151	155	(66.5)	31	(75.6)	0.251	162	(66.9)	24	(75.0)	0.359
Posttraumatic stress reactions (mean PTSD-RI)	Wave 1	1.53	(0.68)	1.64	(0.78)	0.202	1.51	(0.70)	1.86	(0.71)	0.001	1.53	(0.71)	1.82	(0.71)	0.023
	Wave 2	1.21	(0.66)	1.33	(0.74)	0.174	1.18	(0.67)	1.60	(0.71)	<0.001	1.20	(0.67)	1.60	(0.74)	0.002
Anxiety/depression symptoms (mean SCL-8)	Wave 1	2.04	(0.63)	2.13	(0.71)	0.259	2.03	(0.66)	2.28	(0.62)	0.015	2.04	(0.66)	2.26	(0.63)	0.067
	Wave 2	1.79	(0.64)	1.83	(0.70)	0.609	1.74	(0.63)	2.12	(0.71)	<0.001	1.77	(0.65)	2.06	(0.69)	0.015
Somatic symptoms (mean CSSI-8)	Wave 1	1.67	(0.51)	1.82	(0.58)	0.016	1.69	(0.54)	1.89	(0.54)	0.017	1.70	(0.54)	1.87	(0.53)	0.075
	Wave 2	1.60	(0.50)	1.72	(0.53)	0.069	1.61	(0.51)	1.81	(0.51)	0.015	1.62	(0.51)	1.80	(0.52)	0.050
Social support (mean FSSQ-7)	Wave 1	4.65	(0.45)	4.37	(0.75)	0.001	4.63	(0.52)	4.18	(0.73)	<0.001	4.61	(0.53)	4.16	(0.76)	0.001
	Wave 2	4.62	(0.49)	4.43	(0.76)	0.035	4.60	(0.55)	4.33	(0.80)	0.040	4.59	(0.55)	4.34	(0.87)	0.123

## Results

**Fig. 1:**
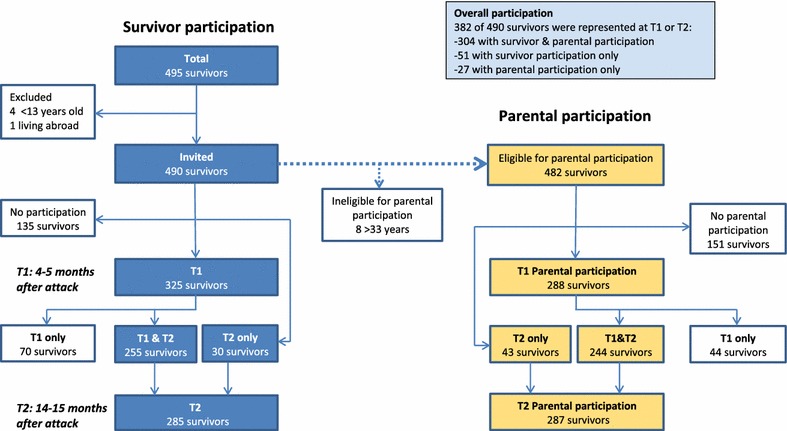
Flow chart of the study participation among survivors of the Utøya shooting (on the *left*) and survivors with parental participation (on the *right*). In total, 355 of the 490 (72 %) invited survivors participated in one or both study waves, and 331 of the 482 (69 %) survivors aged <33 years had ≥1 parent(s) who participated in the study

Altogether, 355 of 490 (72.4 %) survivors participated in the study, including 325 (66.3 %) in wave 1 and 285 (58.2 %) in wave 2. In wave 1, three survivors opted out when they received the invitation letter by sending a text message to the research team. In addition, 29 survivors could not be reached by telephone. In wave 2, seven survivors opted out before they were called, and 43 could not be reached by telephone. In wave 1, survivors were interviewed from 2 November 2011 to 5 March 2012, including >95 % in November and December. In wave 2, the survivors were interviewed from 13 September 2012 to 5 February 2013, including 90 % during the first 2 months. Figure 1 illustrates the survivor participation and parental participation by study wave. Overall, 255 (52.0 %) survivors participated in both waves: 70 (14.3 %) in wave 1 only and 30 (6.1 %) in wave 2 only. In total, 331 of 482 (68.7 %) survivors who were eligible for parental inclusion had ≥1 parents in the parental study, including 304 participating and 27 non-participating survivors. Therefore, we obtained data from either the survivor or the parents for 382 (78.0 %) survivors. There were 223 survivors with both maternal and paternal participation, 88 with only maternal participation, and 20 with only paternal participation. Altogether, 531 caregivers participated in ≥1 waves: 299 female (291 mothers, six stepmothers/foster mothers and two other female relatives) and 232 male caregivers (216 fathers and 16 stepfathers/foster fathers). Five survivors were represented by two female caregivers, i.e., a mother and a stepmother (n = 3) or other female relative (n = 2), and four survivors were represented by two male caregivers, i.e., a father and a stepfather. Because we examined parental participation, we excluded the two other female relatives.

Non-participating survivors did not significantly differ from those who participated in ≥1 waves with respect to age, gender, hospitalization or geographical region of residence, although a nearly significantly (p = 0.055) larger proportion of participants were female (Table 1). Compared with survivors who participated in both waves, those who only participated in wave 1 were more likely to be non-Norwegian, non-members of the youth Labour Party and to report higher levels of terror exposure. Those who only participated in wave 2 reported more posttraumatic stress reactions, anxiety/depression symptoms, and somatic symptoms (Table 2).

Table 3 presents factors associated with parental participation among the participating survivors. Altogether, 304 of 348 (87.4 %) participating survivors who were eligible for parental inclusion had ≥1 parents in the study: 289 with maternal and 228 with paternal participation. They represented 286 families: 270 families with one, 14 families with two, and two families with three participating survivors (i.e., siblings). Parental non-participation was associated with the following survivor characteristics: higher age; non-Norwegian origin; not living with parents; not having siblings in the study; and more posttraumatic stress reactions, anxiety/depression symptoms, somatic symptoms, and less social support. The same associations applied for maternal non-participation, except for not having siblings in the study. Paternal non-participation was associated with non-Norwegian origin, divorced parents, not having siblings in the study, somatic symptoms and less social support. Additionally, survivors with paternal non-participation in wave 1 were more likely to have higher levels of posttraumatic stress and anxiety/depression symptoms in wave 2. Moreover, survivors without maternal or paternal participation in wave 1 were more likely to use mental health services in wave 2 (Additional file 1: Appendix 1, Additional file 2: Appendix 2). A supplementary analysis of only survivors without siblings in the study (n = 314) yielded a similar pattern of associations. No maternal participation in wave 1 was associated with a loss of follow-up: 40 (16.0 %) of the survivors who participated in both waves had no maternal participation in the first wave versus 19 (27.5 %) of those who only participated in wave 1. No such associations were found with respect to paternal or any parental participation.

## Discussion

The Utøya attack was a severe trauma that resulted in high levels of posttraumatic distress and extensive health-care utilization among survivors [12, 19]. After the attack, the survivors were geographically dispersed, and many soon relocated to start their studies. Despite these potentially unfavourable research conditions, nearly three of every four survivors participated in at least one survey wave. Therefore, satisfactory response rates can be obtained in studies launched after terrorism incidents. Longitudinal studies are essential to increase our understanding of how terrorism affects survivor health over time. However, our findings indicate that survivors who participate longitudinally differ from those who only participate once. Compared with survivors who participated in both survey waves, those who were lost to follow-up after wave 1 were more likely to be non-Norwegian and non-members of the political youth organization and to report higher levels of exposure. In contrast, the survivors who entered the study at wave 2 reported more posttraumatic stress reactions, symptoms of anxiety/depression and somatic symptoms. Therefore, the exclusion of survivors who do not participate longitudinally might increase the likelihood of selection bias. This would most likely lead to an underestimation of the impact of the disaster, since the levels of exposure and symptoms were higher among survivors who did not participate in both waves. This possibility can be counteracted by collecting longitudinal register-based data on, e.g., socioeconomics and health-care utilization for all participants. If possible, one should therefore consider requesting consent for data linkage when participants join the study. Another approach to account for attrition is the use of statistical methods, such as multiple imputation [20].

Former longitudinal post-disaster studies have generally consisted of closed cohorts, in which only subjects who participated in the first assessment were invited to participate in ensuing assessments. The open cohort design of our study enabled survivors to join the study at wave 2 despite initial non-participation. This approach might have improved the response rate among survivors who were unable to participate directly after the event. The elevated symptom levels of survivors who entered our study at wave 2 might indicate that an open cohort design facilitates the inclusion of survivors who may have been unable to participate initially because of adverse health consequences (Table 2). Therefore, an open cohort might yield a more representative sample. However, survivors who participated in wave 1 may also have been more likely to receive care than non-participants if the interviewers identified unmet needs, which may have contributed to lower symptom levels in the survivors who participated in wave 1.

Our study supports previous findings that indicate lower response rates among immigrant survivors [4, 5]. These survivors were more likely to be lost to follow-up and less likely to have parents who participated in the study. These results suggest that additional attention should be paid to the recruitment of immigrants. A longitudinal study on survivors of a fireworks disaster in the Netherlands also found that the overall response rate was lower among immigrants [4]. Additionally, the response patterns differed between immigrants and native Dutch survivors. Whereas health problems were associated with higher response among immigrant survivors, health problems were associated with lower response among non-immigrants. Therefore, the risk of selection bias might be higher for findings related to immigrant status and should be considered in the interpretation of findings.

Although the results vary between studies, research respondents typically have higher socioeconomic status and better health than non-respondents [5, 21, 22]. We did not find significant differences between participants and non-participants with respect to age, gender, centrality of residence, or hospitalization (Table 1). However, for non-participants, we lacked information regarding the factors that differed between survivors who participated in both waves and one wave only (Table 2). Therefore, it remains uncertain whether non-participants differed from participants with respect to ethnicity, terror exposure and health characteristics.

Findings on factors associated with research participation among disaster survivors are conflicting. In certain post-disaster studies, non-participation has been associated with sociodemographic factors (such as being male, unmarried, and having low income) and health-related factors (such as symptoms of PTSD and depression) [23, 24]. Other studies have not found such associations [25, 26]. The results on exposure are also inconsistent. One study found that exposure was related to attrition [27], whereas another found that a threat to life was associated with follow-up participation [28].

Factors associated with research participation might also differ by groups of survivors and study method. Because the survivors in our study were confined to an islet during the attack, they may have been easier to identify than survivors of disasters without distinct geographical boundaries. Additionally, most of the survivors were young members of the same political youth party. This shared affiliation might have facilitated the dissemination of study information and motivated the survivors to participate. In fact, members were more likely to participate in both waves than non-members (Table 2). It is also possible that the survivors of the Utøya attack, who were mostly politically active youth, were socioeconomically more homogenous than a random population-based sample, which might have contributed to the absence of sociodemographic differences with respect to participation except ethnicity. The interview experience of the participants during wave 1 might also have influenced subsequent participation. We lacked information regarding how the respondents experienced the interview. However, it has been demonstrated that a negative experience of an interview is associated with attrition [21].

Our study was based on in-depth interviews performed across the entire country and was consequently highly demanding of resources. In addition to the research data that were collected, the health practitioners received up-to-date information on treating trauma, and the researchers gained insight into the challenges that health practitioners face when they met survivors. This integration of research and clinical practice might strengthen the understanding of trauma among clinicians and researchers, and be valuable in future research. Although our study may not be generalizable to low-resource settings, the integration of research and trauma education could be particularly important in low-income countries, where the risk of disasters is highest and there is often a lack of trained personnel [29].

Population-based health studies on adolescents and young adults evince an overrepresentation of youth raised by two parents and from families with high income and education [30]. We did not find significant differences between the survivors who participated in both waves and those who participated in only one wave with respect to divorced parents or self-perceived financial status. However, we lacked such information for non-participants.

Regarding parental participation, findings from family studies indicate that fewer fathers than mothers participate in research [31]. This finding agrees with our results. However, in our study, the difference was less pronounced, which might reflect that Norway is a country with relatively high gender equality. Thus, fathers might be more involved in the upbringing of their children compared with countries with less gender equality. Alternative explanations might be that fathers are more inclined to participate after catastrophic events than in everyday settings or that our study method was more successful in reaching fathers, e.g., flexibility with respect to the interview time and location. Parental non-participation was associated with non-Norwegian ethnicity, somatic symptoms and less social support. Otherwise, factors associated with maternal and paternal participation diverged (Table 3). Paternal non-participation was associated with having divorced parents and not having siblings in the study, whereas maternal non-participation was associated with higher age, not living with parents, and higher levels of posttraumatic stress reactions and anxiety/depressions symptoms. Therefore, it is important to consider potential differential selection of mothers and father in the interpretation of parental data. For instance, a divorce might influence the health of both youth and their parents. Survivors were less likely to have participating fathers if the parents were divorced, whereas a nearly significantly larger proportion of survivors with participating mothers had divorced parents. Prior research that involved parents also indicated that well-functioning fathers with high socioeconomic status tend to be overrepresented [31]. We obtained parent contact information from the survivors. Therefore, whether a parent was invited to participate in the study may have depended on the survivor. Thus, non-response among parents could reflect a weaker parent-offspring relationship.

Non-participation does not necessarily result in bias. However, the estimated associations may be biased if non-participation is related to the severity of the outcome and/or the exposure under study [32]. Therefore, non-response and attrition should be comprehensively assessed because they might threaten the validity of a study.

### Strengths and limitations

This study provides new data with respect to non-participation that can be valuable for planning and interpreting related research. The open cohort design yielded new information regarding survivors who did not participate in the first survey wave conducted soon after the attack but joined the study later. In previous studies, which generally use closed cohorts, these survivors would have been non-participants. Prior post-disaster studies have also commonly lacked a clear definition of the study population, whereas our study population was clearly defined by the geographical constriction of the island. Past studies on children and adolescents have often only collected data from parents and included only one parent, typically the mother [3]. We obtained data from adolescents and parents, who represented directly (i.e., were present during the attack) and indirectly (i.e., had children who were at risk of being killed during the attack) exposed samples, respectively. In addition, our study included maternal and paternal reports for most of the survivors. Moreover, the study included in-depth interviews with little missing data.

The study also had several limitations. The study did not provide active intervention or treatment. However if unmet needs were revealed during wave 1, the interviewers were recommended to assist the participants in acquiring suitable care. Therefore, wave 1 participants may have been more likely to receive timely support than non-participants because of study participation, which may have contributed to the lower levels of symptoms in survivors who participated in both waves compared with those who only participated in wave 2. Furthermore, in the preparation of the study, we endeavored to design the questionnaires to suit both adolescents and young adults. Nevertheless, the age range of survivors might have led to variation in the ability to respond to the questionnaire. In addition, we do not know how many parents were invited to participate because their contact information was acquired from the survivors. This fact could have increased the likelihood of selection bias with respect to parental participation. There might also have been variation in the efforts of the interviewers to acquire contact information for both parents. In the instructions to the interviewers, the wording on parental invitation for survivors aged less than 16 years differed slightly from that for those aged ≥16 years. The interviewers were requested to ask survivors under 16 years old for the telephone number of one of the parents to request consent for their offspring’s participation. In contrast, they were requested to ask those aged ≥16 years for contact information for both parents. This approach might explain why only half of the survivors under 16 years old had at least two parents in the study, whereas two-thirds of survivors aged ≥16 years had two or more parents in the study (data not shown). Because there were only 29 survivors aged <16 years in our study, this circumstance is unlikely to have substantially affected the results. We had little data on non-participants and lacked information regarding ethnicity, exposure, and symptoms among non-participants. These factors were associated with loss of follow-up or initial non-participation, and we cannot determine whether the same associations applied for non-participation in both waves. Additionally, the study was based on self-reports and lacked pre-disaster data. Finally, our analysis might have failed to detect significant differences because of a relatively small sample size (i.e., type II error) (Additional file 3).

## Conclusions

Compared with survivors who participated longitudinally, those survivors who were lost to follow-up after wave 1 were more likely to be non-Norwegian and report higher exposure. In contrast, those survivors who entered the study at wave 2 reported more posttraumatic stress, anxiety/depression, and somatic symptoms. Therefore, it is beneficial to avoid exclusion of participants who do not participate longitudinally, for instance, by collecting longitudinal data through a linkage to registers. In addition, an open cohort design that enables survivors to join the study in a later stage despite initial non-participation might improve the response rate among survivors with increased symptoms levels and yield a more representative sample. Finally, the factors associated with maternal and paternal participation differed. Parental non-participation was associated with non-Norwegian origin, somatic symptoms and less social support. Additionally, paternal non-participation was associated with having divorced parents, and maternal non-participation was associated with higher age, not living with parents, more posttraumatic stress and anxiety/depression symptoms. Consequently, it is important to consider a potential differential sample selection of mothers and fathers in the analysis and interpretation of the parental data.

## Additional files

10.1186/s13104-016-1873-1 Survivor characteristics by maternal and paternal participation in wave 1 among survivors aged 13–32 years who participated in wave 1 or 2 (n = 348).
10.1186/s13104-016-1873-1 Survivor characteristics by maternal and paternal participation in wave 2 among survivors aged 13–32 years who participated in wave 1 or 2 (n = 348).
10.1186/s13104-016-1873-1 STROBE checklist.
