# Supplementary material for: Research participation after terrorism: an open cohort study of survivors and parents after the 2011 Utøya attack in Norway
Source: BMC Res Notes. 2016 Feb 1;9:57. doi: 10.1186/s13104-016-1873-1 (PMC4736239; doi:10.1186/s13104-016-1873-1)
Supplement: Supplementary file 1 — 10.1186/s13104-016-1873-1 Survivor characteristics by maternal and paternal participation in wave 1 among survivors aged 13–32 years who participated in wave 1 or 2 (n = 348). [file 13104_2016_1873_MOESM1_ESM.pdf]

Appendix 1.

Survivor characteristics by maternal and paternal participation in wave 1 among survivors aged 13 -32 years who participated in wave 1 or 2 (n=348).

		**Paternal participation wave 1**					**Maternal participation wave 1**					**Any parental participation wave 1**				
		Yes (n=201)		No (n=147)			Yes (n=261)		No (n=87)			Yes (n=278)		No (n=70)		
**Survivor characteristics**		n/mean	(%/sd)	n/mean	(%/sd)	p-value	n/mean	(%/sd)	n/mean	(%/sd)	p-value	n/mean	(%/sd)	n/mean	(%/sd)	p-value
Mean age in years		18.64	(2.63)	19.20	(3.76)	0.120	18.61	(2.86)	19.67	(3.87)	0.021	18.63	(2.85)	19.84	(4.09)	0.021
Male gender		105	(52.2)	76	(51.7)	0.921	137	(52.5)	44	(50.6)	0.757	144	(51.8)	37	(52.9)	0.874
Non-Norwegian origin		14	(7.0)	25	(17.6)	0.002	17	(6.5)	22	(26.8)	<0.001	21	(7.6)	18	(27.7)	<0.001
Financially disadvantaged		38	(19.5)	33	(23.2)	0.404	48	(19.0)	23	(27.4)	0.102	52	(19.3)	19	(28.4)	0.102
Divorced parents (Wave 1)		64	(32.3)	61	(55.0)	<0.001	106	(41.7)	19	(34.5)	0.325	113	(41.7)	12	(31.6)	0.234
Living with ≥1 parents (Wave 1)		131	(66.8)	71	(60.2)	0.232	177	(69.1)	25	(43.1)	<0.001	187	(68.5)	15	(36.6)	<0.001
Sibling(s) in the study		25	(12.4)	9	(6.1)	0.050	27	(10.3)	7	(8.0)	0.532	32	(11.5)	2	(2.9)	0.029
Hospitalized		13	(6.5)	15	(10.2)	0.206	21	(8.0)	7	(8.0)	1.000	23	(8.3)	5	(7.1)	0.756
Terror exposure (mean 0-13)		8.41	(2.26)	8.77	(2.14)	0.144	8.51	(2.16)	8.72	(2.39)	0.453	8.47	(2.17)	8.91	(2.37)	0.150
Mental health service	Wave 1	142	(71.7)	86	(74.1)	0.642	183	(71.8)	45	(76.3)	0.484	197	(72.4)	31	(73.8)	0.852
utilization	Wave 2	100	(62.9)	86	(74.8)	0.038	133	(63.9)	53	(80.3)	0.013	141	(64.7)	45	(80.4)	0.025
Posttraumatic stress reactions	Wave 1	1.53	(0.67)	1.62	(0.79)	0.338	1.51	(0.71)	1.80	(0.70)	0.004	1.53	(0.71)	1.79	(0.71)	0.029
(mean PTSD-RI)	Wave 2	1.16	(0.62)	1.37	0.77	0.013	1.14	(0.65)	1.56	(0.72)	<0.001	1.16	(0.65)	1.57	(0.75)	<0.001
Anxiety/depression symptoms	Wave 1	2.05	(0.64)	2.10	(0.69)	0.450	2.03	(0.66)	2.24	(0.61)	0.024	2.04	(0.66)	2.22	(0.64)	0.107
(mean SCL-8)	Wave 2	1.73	(0.59)	1.90	(0.74)	0.035	1.70	(0.60)	2.13	(0.72)	<0.001	1.72	(0.62)	2.12	(0.73)	<0.001
Somatic symptoms	Wave 1	1.68	(0.51)	1.79	(0.58)	0.063	1.69	(0.54)	1.86	(0.54)	0.028	1.70	(0.54)	1.85	(0.53)	0.085
(mean CSSI-8)	Wave 2	1.56	(0.44)	1.75	(0.59)	0.004	1.58	(0.47)	1.83	(0.61)	0.002	1.58	(0.47)	1.84	(0.62)	0.004
Social support	Wave 1	4.64	(0.45)	4.43	(0.72)	0.006	4.63	(0.53)	4.25	(0.69)	<0.001	4.61	(0.54)	4.24	(0.73)	0.003
(mean FSSQ-7)	Wave 2	4.61	(0.49)	4.48	(0.72)	0.086	4.61	(0.53)	4.39	(0.76)	0.027	4.60	(0.54)	4.41	(0.78)	0.095
