# Supplementary material for: Research participation after terrorism: an open cohort study of survivors and parents after the 2011 Utøya attack in Norway
Source: BMC Res Notes. 2016 Feb 1;9:57. doi: 10.1186/s13104-016-1873-1 (PMC4736239; doi:10.1186/s13104-016-1873-1)
Supplement: Supplementary file 2 — 10.1186/s13104-016-1873-1 Survivor characteristics by maternal and paternal participation in wave 2 among survivors aged 13–32 years who participated in wave 1 or 2 (n = 348). [file 13104_2016_1873_MOESM2_ESM.pdf]

Appendix 2.

Survivor characteristics by maternal and paternal participation in wave 2 among survivors aged 13 -32 years who participated in wave 1 or 2 (n=348).

		**Paternal participation wave 2**					**Maternal participation wave 2**					**Any parental participation wave 2**				
		Yes (n=166)		No (n=182)			Yes (n=243)		No (n=105)			Yes (n=262)		No (n=86)		
**Survivor characteristics**		n/mean	(%/sd)	n/mean	(%/sd)	p-value	n/mean	(%/sd)	n/mean	(%/sd)	p-value	n/mean	(%/sd)	n/mean	(%/sd)	p-value
Mean age in years		18.92	(2.88)	18.83	(3.41)	0.788	18.63	(2.92)	19.44	(3.62)	0.028	18.65	(2.89)	19.58	(3.83)	0.043
Male gender		90	(54.2)	91	(50.0)	0.432	127	(52.3)	54	(51.4)	0.886	138	(52.7)	43	(50.0)	0.667
Non-Norwegian origin		6	(3.6)	33	(18.6)	<0.001	15	(6.2)	24	(24.0)	<0.001	16	(6.1)	23	(28.4)	<0.001
Financially disadvantaged		30	(18.8)	41	(23.2)	0.321	46	(19.4)	25	(25.0)	0.250	50	(19.7)	21	(25.3)	0.276
Divorced parents (wave 1)		42	(28.0)	83	(52.2)	<0.001	96	(43.6)	29	(32.6)	0.073	101	(42.6)	24	(33.3)	0.160
Living with ≥1 parents (wave 1)		97	(62.8)	105	(64.8)	0.853	151	(67.7)	51	(56.0)	0.050	161	(67.1)	41	(55.4)	0.067
Sibling(s) in the study		18	(10.8)	16	(8.8)	0.520	27	(11.1)	7	(6.7)	0.200	29	(11.1)	5	(5.8)	0.154
Hospitalized		11	(6.6)	17	(9.3)	0.353	19	(7.8)	9	(8.6)	0.813	21	(8.0)	7	(8.1)	0.971
Terror exposure (mean 0-13)		8.50	(2.25)	8.61	(2.18)	0.652	8.54	(2.15)	8.59	(2.36)	0.842	8.51	(2.16)	8.71	(2.37)	0.483
Mental health service	Wave 1	103	(68.7)	125	(76.2)	0.134	159	(72.3)	69	(73.4)	0.837	169	(71.3)	59	(76.6)	0.364
utilization	Wave 2	96	(64.9)	90	(71.4)	0.246	137	(65.2)	49	(76.6)	0.089	149	(65.9)	37	(77.1)	0.133
Posttraumatic stress reactions	Wave 1	1.50	(0.67)	1.63	(0.75)	0.106	1.48	(0.70)	1.77	(0.70)	0.001	1.50	(0.70)	1.78	(0.72)	0.002
(mean PTSD-RI)	Wave 2	1.22	(0.67)	1.27	(0.71)	0.546	1.15	(0.65)	1.56	(0.72)	<0.001	1.17	(0.65)	1.59	(0.76)	<0.001
Anxiety/depression symptoms	Wave 1	2.02	(0.64)	2.11	(0.67)	0.260	2.00	(0.65)	2.23	(0.65)	0.003	2.00	(0.65)	2.26	(0.64)	0.002
(mean SCL-8)	Wave 2	1.81	(0.65)	1.79	(0.67)	0.802	1.71	(0.61)	2.08	(0.74)	<0.001	1.74	(0.63)	2.10	(0.72)	0.002
Somatic symptoms	Wave 1	1.65	(0.49)	1.79	(0.58)	0.023	1.68	(0.52)	1.80	(0.58)	0.073	1.68	(0.52)	1.85	(0.58)	0.008
(mean CSSI-8)	Wave 2	1.60	(0.53)	1.68	(0.49)	0.172	1.58	(0.49)	1.81	(0.55)	0.002	1.60	(0.49)	1.83	(0.57)	0.003
Social support	Wave 1	4.66	(0.44)	4.47	(0.67)	0.002	4.62	(0.53)	4.42	(0.66)	0.007	4.63	(0.52)	4.35	(0.69)	0.001
(mean FSSQ-7)	Wave 2	4.64	(0.46)	4.46	(0.72)	0.013	4.60	(0.55)	4.41	(0.73)	0.054	4.59	(0.55)	4.39	(0.78)	0.068
